# Supplementary material for: Astaxanthin Prevents Atrophy in Slow Muscle Fibers by Inhibiting Mitochondrial Reactive Oxygen Species via a Mitochondria-Mediated Apoptosis Pathway
Source: Nutrients. 2021 Jan 26;13(2):379. doi: 10.3390/nu13020379 (PMC7912339; doi:10.3390/nu13020379)
Supplement: Supplementary file 1 [file nutrients-13-00379-s001.pdf]

Supplementary Table S1 Composition of the experimental diets

	ND	AX
α-corn starch (g)	45.4	45.4
Sucrose (g)	22.8	22.8
Casein (g)	20.0	20.0
Cystine (g)	0.3	0.3
Soybean oil (g)	5.0	4.8
Bio Astin SCE (g)	0	0.2
Vitamin mix (g)	1.0	1.0
Mineral mix including choline (g)	3.5	3.5
Cellulose (g)	2.0	2.0
Tertiary butylhydroquinone (g)	0.0014	0.0014

Bio Astin SCE contains 10% astaxanthin derived from *Haematococcus pluvialis*. ND, normal diet; AX, astaxanthin-supplemented diet.

Supplementary Table S2 Primer sets used in this study

Target gene		Sequence
AMPK alpha-1	F	5’- TCAGTTCCTGGAGAAAGATGG-3’
	R	5’- TTATGTCCGGTCAACTCGTG-3’
PPAR gamma	F	5’- CCCATCGAGGACATCCAA-3’
	R	5’- CACGTGCTCTGTGACGATCT-3’
Ckmt 2	F	5’- TACTCACGGGCAGTTTGATA-3’
	R	5’- CACATTCTCCACCTCCCTTC-3’
Ucp2	F	5’- GCGTTCTGGGTACCATCCTA-3’
	R	5’- AGAGTCGTAGAGGCCAATGC-3’
Atp5g1	F	5’- CCATCTAAGCAGCCTTCCTG-3’
	R	5’- GATCCAGCCACACCAACTGT-3’
Ndufaf2	F	5’- AGGCATGAGCTGGTGGTC-3’
	R	5’- TCTGCCCTCTCCAGTTCTTG-3’
Sdhb	F	5’- GGAGGGCAAGCAACAGTATC-3’
	R	5’- CTTGTCTCCGTTCCACCAGT-3’
GAPDH	F	5’- ACCCAGAAGACTGTGGATGG -3’
	R	5’- TTCAGCTCTGGGATGACCTT - 3’

F, forward primer; R, reversed primer; AMPK, Adenosine 5‘-monophosphate (AMP)-activated protein kinase; PPAR, peroxisome proliferator-activated receptor; Ckmt, creatine kinase in mitochondrial; UCP, uncoupling protein; Atp5g1, ATP synthase, H^+ transporting, mitochondrial F0 complex, subunit C1; Ndufaf2, NADH-ubiquinone oxidoreductase complex assembly factor; Sdhb, succinate dehydrogenase complex, subunit B; GAPDH, glycelaldehyde-3-phosphate dehydrogenase.
